# CircZBTB46 alleviates metabolic dysfunction–associated steatotic liver disease by targeting miRNA-326/FGF1 axis

**DOI:** 10.1038/s41420-025-02833-x

**Published:** 2026-01-09

**Authors:** Qing-Min Zeng, Tengyue Hu, Wei Jiang, Xiangnan Teng, Dongbo Wu, Hong Tang, Chang-Hai Liu

**Affiliations:** 1 Center of Infectious Diseases, West China Hospital, Sichuan University, Chengdu, China; 2 Laboratory of Infectious and Liver Diseases, Institute of Infectious Diseases, West China Hospital, Sichuan University, Chengdu, China

**Keywords:** Non-alcoholic fatty liver disease, Non-coding RNAs

## Abstract

Metabolic dysfunction–associated steatotic liver disease (MASLD) is a leading cause of chronic liver disease worldwide, characterized by multiple metabolic disturbances. This complexity poses significant challenges for early diagnosis and effective treatment, highlighting the urgent need for novel biomarkers and therapeutic strategies. Circular RNAs (circRNAs) have attracted attention due to their unique stability and regulatory roles in various diseases, providing new opportunities for MASLD diagnosis and treatment. This study investigated the role of circZBTB46 in MASLD and its underlying molecular mechanism. Liver tissues from three healthy controls, three patients with MASLD, and three patients with metabolic dysfunction–associated steatohepatitis (MASH) were analyzed using RNA sequencing and bioinformatics analysis to identify differentially expressed circRNAs. CircRNA-miRNA interactions were predicted through the circinteractome database and validated by dual-luciferase reporter gene assays and RNA pull-down experiments. mRNA and protein expression were evaluated by qRT-PCR and western blot, while triglyceride and cholesterol levels were measured by ELISA. Lipid deposition was visualized through Oil Red O and BODIPY 493/503 staining. The results showed that circZBTB46, derived from the ZBTB46 gene, was downregulated in patients with MASLD and in experimental models. Overexpression of circZBTB46 significantly reduced hepatic lipid accumulation and triglyceride content. This effect is mediated through the circZBTB46/miRNA-326/FGF1 pathway, in which circZBTB46 directly binds to miRNA-326, functioning as a competitive endogenous RNA (ceRNA) to relieve miRNA-326-mediated suppression of FGF1, thereby alleviating hepatic lipid accumulation. These findings reveal the critical role of circZBTB46 in MASLD and provide valuable insights into its potential as a diagnostic biomarker and therapeutic target for MASLD.

## Introduction

Metabolic dysfunction–associated steatotic liver disease (MASLD), previously known as non-alcoholic fatty liver disease (NAFLD), is characterized by excessive lipid accumulation in hepatocytes. The renaming of NAFLD to MASLD reflects a deeper understanding of the central role of metabolic dysfunction in its progression and emphasizes its broader association with other metabolic syndromes. MASLD has emerged as a significant public health concern, estimated to affect approximately 32.4% of the global population, with its prevalence continuing to rise [1–3]. Currently, liver biopsy remains the gold standard for the diagnosis of MASLD; however, its invasiveness, high cost, and sampling bias limit its widespread application. Consequently, there is an increasing emphasis on the development of non-invasive biomarkers for the early diagnosis, prognostic assessment, and monitoring of diseases. Although progress has been made in understanding the pathophysiology of MASLD, the molecular pathways underlying hepatic lipid deposition remain incompletely elucidated, which hinders our comprehensive understanding of disease progression and impedes the development of effective therapeutic strategies. A significant milestone in MASLD treatment was the recent approval of Resmetirom, the first drug specifically indicated for patients with non-cirrhotic metabolic dysfunction-associated steatohepatitis (MASH) with moderate to advanced liver fibrosis, which inspired further exploration of targeted treatment strategies [4]. Additionally, non-invasive diagnostic models and targeted therapies based on genetic and epigenetic factors have made significant progress, offering new directions for MASLD research and clinical management. Within this advancing field, circular RNA (circRNA), once considered transcriptional byproducts, has emerged as a key molecular player in various diseases, including MASLD [5, 6].

CircRNAs, a distinct class of non-coding RNAs with a covalently closed circular structure, exhibit remarkable stability due to their resistance to exonuclease degradation, distinguishing them from linear RNAs [6]. This stability enables circRNAs to persist in biological fluids such as plasma, saliva, and urine, allowing for non-invasive detection through methods like blood tests [7–9]. Moreover, circRNAs show tissue- and disease-specific expression patterns, making them promising biomarkers for disease diagnosis and prognosis [7, 8]. Their presence within extracellular vesicles further enhances their diagnostic potential, as these vesicles protect circRNAs from degradation and facilitate their transport and detection in body fluids [10]. Our previous review emphasized the significant role of circRNAs in the molecular mechanisms underlying MASLD, with dynamic changes in their expression correlating with disease progression [11, 12]. CircRNAs act as competitive endogenous RNAs (ceRNAs) that interact with microRNAs (miRNAs) to modulate gene expression [11]. This mechanism emphasizes their significant roles in metabolic regulatory networks and highlights their potential as therapeutic targets for MASLD [7]. However, the exact mechanisms by which circRNAs influence hepatic lipid metabolism remain unclear, and the diversity of circRNA molecules complicates the identification of those most impactful on disease progression. Further validation in large cohort studies and the development of accurate circRNA detection methods for clinical application are imperative. Our previous transcriptome analyses indicated that the expression level of hsa_circ_0002805 (circZBTB46) is inversely correlated with the severity of MASLD [13]. Additionally, previous studies have identified its high expression in atherosclerosis, in which it participates in disease progression through interactions with the RNA-binding protein hnRNPA2B. These findings suggest that circZBTB46 plays a significant role in metabolic dysfunction. Based on these observations, this study aimed to investigate its functional role in MASLD pathology through in vitro and in vivo models. By identifying the specific miRNAs targeted by circZBTB46 and their downstream effects, we aimed to uncover novel insights into the regulation of lipid metabolism in MASLD, thereby revealing its potential application in disease diagnosis and treatment.

Fibroblast growth factor 1 (FGF1), a key member of the fibroblast growth factor family, is critical for adipose tissue remodeling under dietary stress conditions [14]. Recent studies on recombinant FGF1 (rFGF1) have shown promising effects in reducing hyperglycemia, enhancing insulin sensitivity, and alleviating steatosis in animal models [14]. Notably, rFGF1 has been shown to reverse hepatic steatosis and MASH by activating pathways such as AMP-activated protein kinase (AMPK), providing a potential mechanism for its anti-steatotic effects [15]. However, the expression and regulatory dynamics of FGF1 in MASLD remain poorly understood, representing a significant knowledge gap. This study aimed to explore the role of circZBTB46 in MASLD and investigate how circZBTB46-mediated modulation of FGF1 expression influences hepatic lipid deposition.

## RESULTS

### CircZBTB46 was downregulated in MASLD

**Fig. 1:**
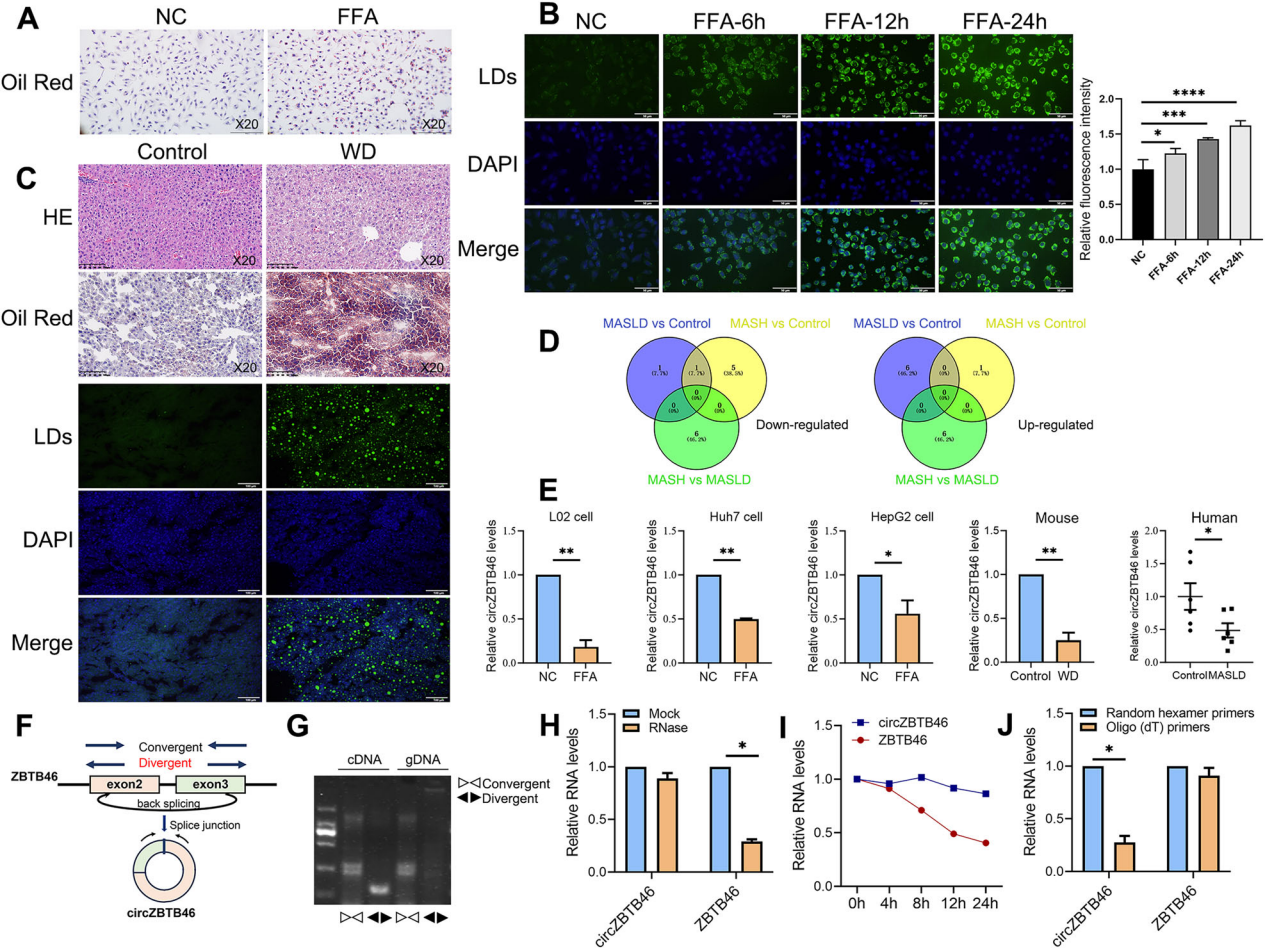
CircZBTB46 expression is decreased in MASLD. **A** Human L02 hepatocytes were incubated with free fatty acids (FFAs) for 12 h, followed by Oil Red O staining to visualize lipid droplet accumulation under a light microscope. Magnification, 20×. **B** L02 cells were incubated with FFAs for 0, 6, 12, and 24 h, and stained with BODIPY 493/503 for lipid droplets (green) and DAPI for nuclei (blue). Scale bars, 50 μm. Fluorescence microscopy showed time-dependent lipid droplet accumulation, with fluorescence intensity quantified using ImageJ software. **C** Representative liver images from a mouse MASLD model established by feeding a high-fat, high-sugar, and high-cholesterol western diet (WD) for 8 weeks. Hematoxylin and eosin (H&E, 20×), Oil Red O (20×), and BODIPY 493/503 (scale bars = 100 μm) staining were used to assess histological changes and lipid accumulation. **D** Venn diagram illustrates the differentially upregulated and downregulated circRNAs identified through high-throughput sequencing of liver tissues from three healthy individuals, three MASLD patients, and three MASH patients. **E** qRT-PCR was performed to assess circZBTB46 expression in liver tissues from both human MASLD patients and mouse models, as well as in FFA-induced cell models. **F** The genomic locus of circZBTB46 is illustrated to provide context for its location within the genome. **G** PCR amplification of cDNA or genomic DNA was performed using divergent and convergent primers, with agarose gel electrophoresis of the PCR products confirming the circularization of circZBTB46. **H** qRT–PCR analysis assessed circZBTB46 and ZBTB46 mRNA expression after treatment with RNase R in L02 cells, indicating the stability of circZBTB46 compared to linear RNAs. **I** qRT–PCR analysis assessed circZBTB46 and ZBTB46 mRNA expression after treatment with Actinomycin D at the indicated time points in L02 cells, revealing the stability of circZBTB46 over time. **J** qRT–PCR analysis assessed circZBTB46 and ZBTB46 mRNA expression using Random hexamer and oligo (dT)18 primer, indicating circZBTB46 does not possess a poly(A) tail. Data represent mean ± S.D. The P value was determined by a two-tailed unpaired Student’s t-test. *P < 0.05, **P < 0.01 vs. control.

We utilized an in vitro MASLD cell model stimulated with free fatty acids (FFA) and an in vivo MASLD animal model induced by WD. In vitro, Oil Red O staining (Fig. 1A) and BODIPY 493/503 staining (Fig. 1B) showed a significant increase in hepatic lipid deposition. In vivo, HE staining, Oil Red O staining, and BODIPY 493/503 staining further confirmed a significant increase in intrahepatic lipid accumulation (Fig. 1C). These results indicate successful MASLD modeling. To investigate the role of circRNAs in MASLD, we conducted transcriptome sequencing of liver tissues from three groups: healthy controls, patients with MASLD, and patients with MASH. The results identified circZBTB46 as consistently downregulated in both MASLD and MASH groups (Fig. 1D). Based on these findings, we focused on circZBTB46 for further investigation. To validate its expression in MASLD, we performed qRT-PCR analysis on the FFA-induced MASLD cell model, including L02 cells (0.18 ± 0.08-fold vs. NC, *P* < 0.01), Huh7 cells (0.50 ± 0.01-fold vs. NC, *P* < 0.01), and HepG2 cells (0.56 ± 0.15-fold vs. NC, *P* < 0.05), as well as in MASLD mice (0.25 ± 0.09-fold vs. control, *P* < 0.01) and liver tissues from MASLD patients (0.49 ± 0.26-fold vs. control, *P* < 0.05) (Fig. 1E). The results consistently showed a significant reduction in circZBTB46 expression levels in the MASLD group compared with the control group (Fig. 1E). Therefore, it can be concluded that circZBTB46 is downregulated in MASLD.

CircZBTB46 is formed by the back-splicing of exons 2–3 of its parental gene ZBTB46 and is located on chromosome 20q13.33, with a mature length of 1255 nucleotides (Fig. 1F). To confirm its circular structure, we performed PCR analysis using convergent and divergent primers designed to span the back-splicing junction, followed by agarose gel electrophoresis. The results showed that circZBTB46 was detectable only in cDNA, but not in gDNA (Fig. 1G). To assess the stability of circZBTB46, we performed RNase R digestion and Actinomycin D treatment experiments. The results revealed that circZBTB46 was resistant to RNase R digestion (Fig. 1H) and exhibited greater stability than linear ZBTB46 (Fig. 1I). Moreover, reverse transcription experiments using either random hexamers or oligo (dT)18 primers showed a significant reduction in circZBTB46 expression when oligo (dT)18 primers were used, suggesting the absence of a poly-A tail in circZBTB46 (Fig. 1J). These results suggest that circZBTB46 is a stably expressed circular RNA.

### CircZBTB46 alleviates intrahepatic lipid deposition in MASLD through FGF1/AMPK signaling

**Fig. 2:**
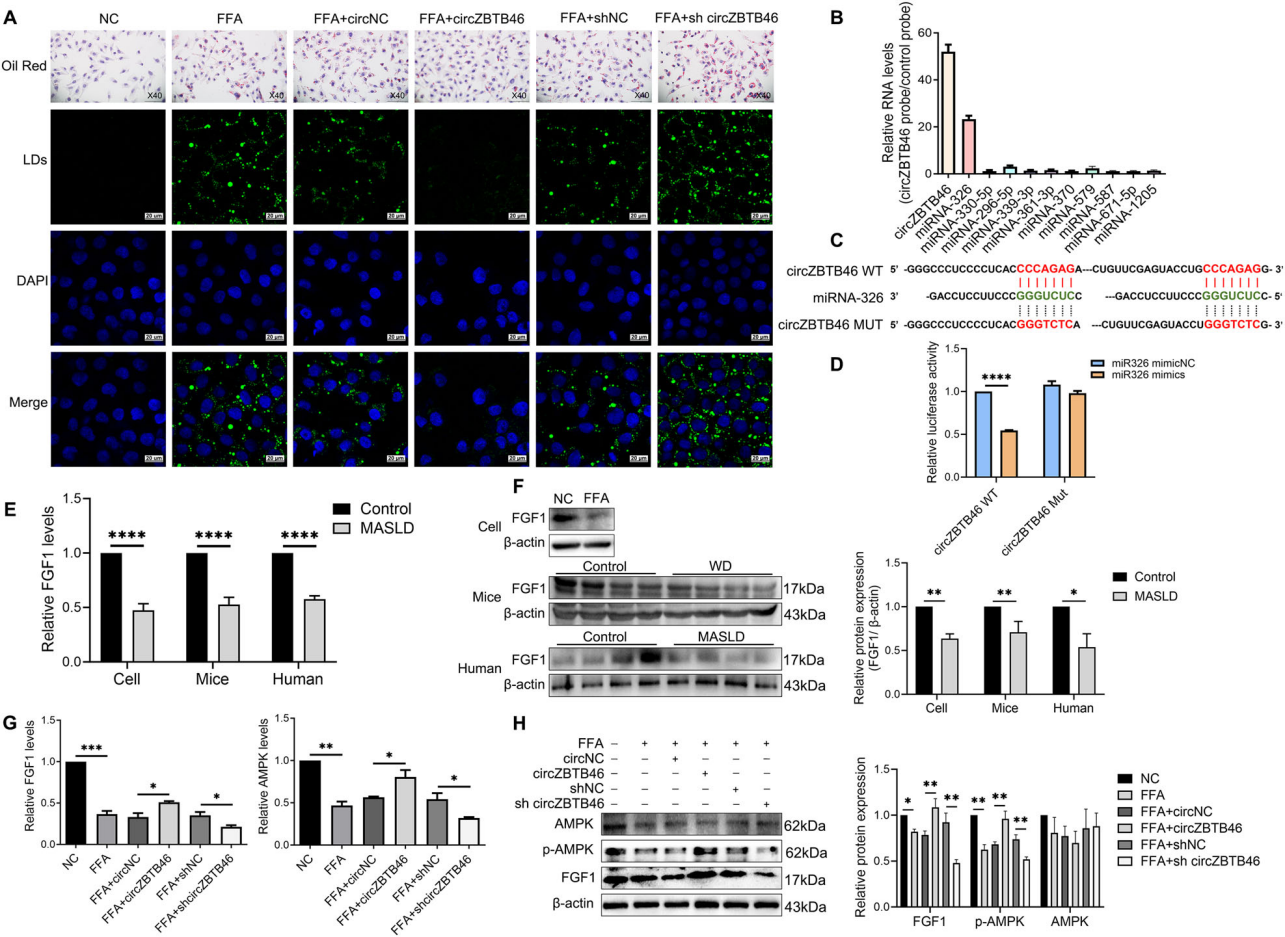
In vitro regulation of intrahepatic lipid accumulation by circZBTB46 via targeting FGF1/AMPK. **A** Representative images of Oil Red O (40×) and BODIPY 493/503 (scale bars = 20 μm) staining in FFA-induced L02 cells transfected with circZBTB46 overexpression or interference plasmids, and control. Lipid droplets (LDs) were stained with BODIPY 493/503 (green), and nuclei were stained with DAPI (blue). **B** Identification of miRNAs pulled down by a circZBTB46 junction probe from RNA extracts of L02 cells. **C** Bioinformatics analysis predicted a potential binding site between circZBTB46 and miRNA-326. **D** Dual luciferase reporter assay showed relative luciferase activity in HEK293T cells co-transfected with miRNA-326 mimics and circZBTB46 reporter plasmid. **E** qRT–PCR analysis assessed FGF1 mRNA expression in FFA-induced L02 cells, a MASLD mouse model, and liver tissues from MASLD patients. **F** Western blot analysis and relative protein quantification were performed to evaluate the expression levels of β-actin and FGF1 in both cell models and liver tissues from MASLD mouse (n = 4) and human (n = 4) samples. **G** qRT–PCR analysis assessed FGF1 and AMPK mRNA expression in FFA-induced L02 cells transfected with circZBTB46 overexpression or interference plasmids, and control plasmids. **H** Western blot analysis and relative protein quantification were assessed for β-actin, FGF1, p-AMPK, and AMPK protein expression in FFA-induced L02 cells transfected with circZBTB46 overexpression or interference plasmids and control plasmids. Relative protein intensities were quantified using ImageJ software. Data represent mean ± S.D. Statistical significance was determined using a two-tailed unpaired Student’s t-test. *P < 0.05, **P < 0.01, ***P < 0.001, ****P < 0.0001 compared to control.

To investigate the role of circZBTB46 in MASLD, we utilized an in vitro MASLD cell model transfected with circZBTB46 overexpression and interference plasmids. Overexpression of circZBTB46 led to a reduction in hepatic lipid levels, as evidenced by decreased intrahepatic lipid deposition observed through Oil Red O staining and a decreased number of lipid droplets within hepatocytes, as shown by BODIPY 493/503 staining, compared to the control group transfected with circNC (Fig. 2A). On the contrary, knockdown of circZBTB46 resulted in increased intrahepatic lipid accumulation (Fig. 2A). These results suggest that circZBTB46 may protect against intrahepatic lipid deposition in MASLD. Considering the role of circRNAs as miRNA sponges, we utilized the circinteractome database (https://circinteractome.nia.nih.gov/) to identify 10 potential miRNAs that could interact with circZBTB46. Among these candidates, the interaction with miRNA-326 was confirmed through RNA pull-down assays (Fig. 2B). Additionally, binding sites between circZBTB46 and miRNA-326 were predicted and validated using dual-luciferase reporter assays (Fig. 2C). The results showed a significant decrease in relative luciferase activity following co-transfection of wild-type (WT) circZBTB46 with an miRNA-326 mimic compared to the negative control (mimicNC) (Fig. 2D). In contrast, no change in luciferase activity was observed when mutated (Mut) circZBTB46 was co-transfected (Fig. 2D). These results suggest an interaction between circZBTB46 and miRNA-326.

To further elucidate the downstream effects of miRNA-326, we explored potential target genes using the miRWalk database (http://mirwalk.umm.uni-heidelberg.de/). Bioinformatic analysis identified FGF1 as the top candidate gene, exhibiting the highest number of potential binding sites and the strongest predicted binding affinity for miRNA-326. To investigate the role of FGF1 in MASLD, we conducted validation experiments to assess FGF1 expression in FFA-induced L02 cells, a MASLD mouse model, and liver tissues from patients with MASLD using qRT-PCR and western blotting. The results revealed a significant decrease in both FGF1 mRNA and protein levels, suggesting a potential involvement of FGF1 dysregulation in the pathogenesis of MASLD (Fig. 2E, F). Building on prior evidence implicating the FGF1/AMPK axis in metabolic disorders, we investigated the impact of circZBTB46 on FGF1 and AMPK. The results indicated that overexpression of circZBTB46 significantly upregulated the mRNA levels of FGF1 and AMPK (both *P* < 0.05), accompanied by an increase in the protein levels of FGF1 and phosphorylated AMPK (p-AMPK), while total AMPK protein levels remained unchanged (Fig. 2G, H). In contrast, circZBTB46 knockdown reversed these effects (Fig. 2G, H). These results suggest that circZBTB46 regulates intrahepatic lipid deposition by modulating the FGF1/AMPK signaling axis, thereby participating in the pathogenesis of MASLD.

### miRNA-326 aggravates intrahepatic lipid deposition in MASLD through FGF1/AMPK signaling

**Fig. 3:**
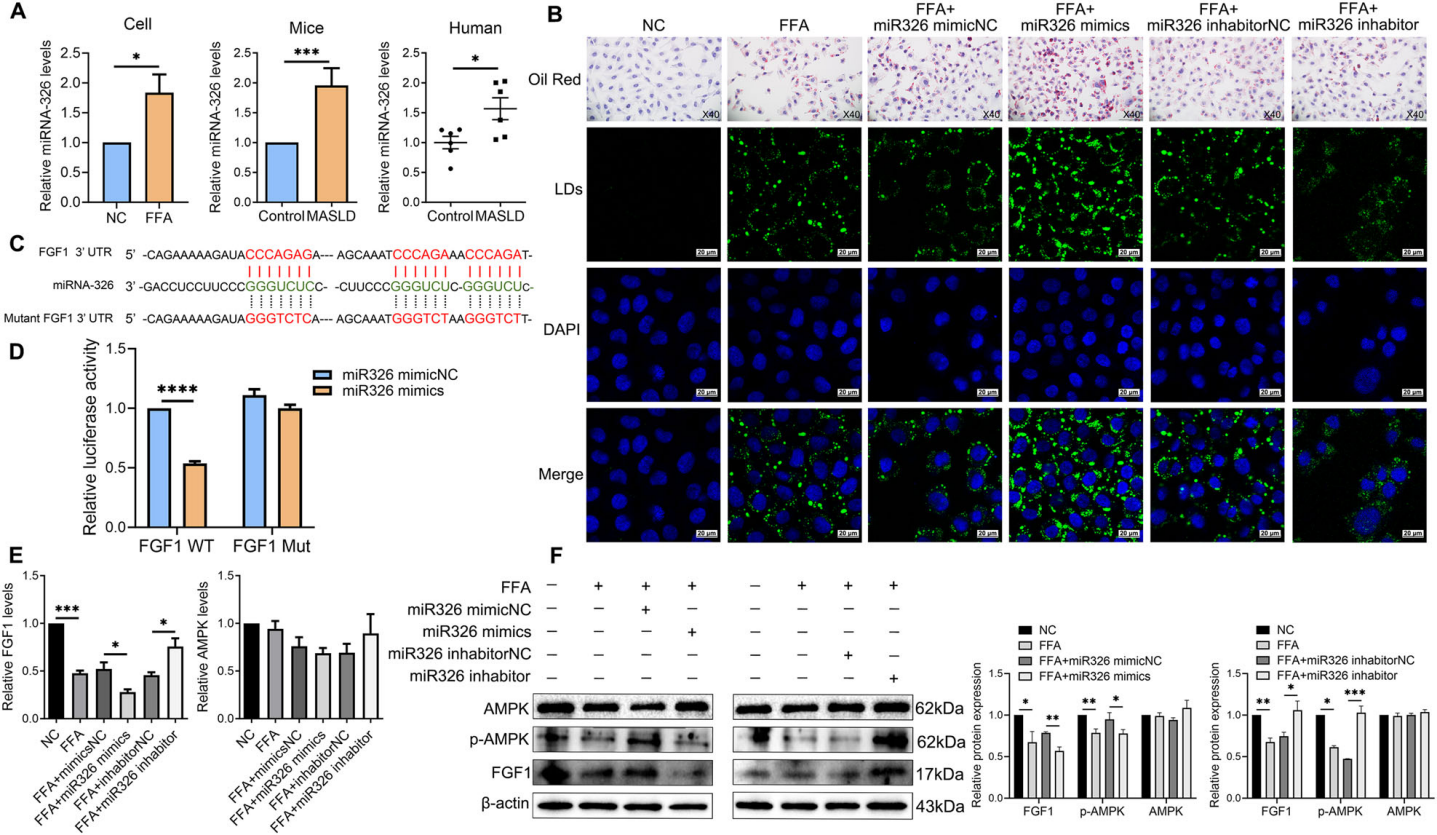
In vitro regulation of intrahepatic lipid accumulation by miRNA-326 via targeting FGF1/AMPK. **A** qRT–PCR analysis assessed miRNA-326 expression in FFA-induced L02 cells, a MASLD mouse model, and liver tissues from MASLD patients. **B** Representative images of Oil Red O (40×) and BODIPY 493/503 (scale bars = 20 µm) staining in FFA-induced L02 cells transfected with miRNA-326 mimics, inhibitors, and control. Lipid droplets (LDs) were stained with BODIPY 493/503 (green), and nuclei were stained with DAPI (blue). **C** Bioinformatics analysis predicted a potential binding site for miRNA-326 in the FGF1 3’-UTR. **D** Dual luciferase reporter assay showed relative luciferase activity in HEK293T cells co-transfected with miRNA-326 mimics and FGF1-WT/MUT 3’-UTR reporter plasmid. **E** qRT–PCR analysis assessed FGF1 and AMPK mRNA expression in FFA-induced L02 cells transfected with miRNA-326 mimics, inhibitors, and controls. **F** Western blot analysis assessed β-actin, FGF1, p-AMPK, and AMPK protein expression in FFA-induced L02 cells transfected with miRNA-326 mimics, inhibitors, and controls. Relative protein intensities were quantified using ImageJ software. Data represent mean ± S.D. Statistical significance was determined using a two-tailed unpaired Student’s t-test. *P < 0.05, **P < 0.01, ***P < 0.001 compared to control.

To investigate the role of miRNA-326 in MASLD, we first quantified its expression in clinical liver tissue samples and established MASLD models. The results showed a significant upregulation of miRNA-326 in both patient samples and MASLD models compared with respective controls (Fig. 3A). We next performed gain and loss-of-function studies by transfecting MASLD cells with miRNA-326 mimics or inhibitors. The results showed that transfection with miRNA-326 mimics significantly increased hepatic lipid levels, as shown by Oil Red O and BODIPY 493/503 staining (Fig. 3B). Conversely, transfection with miRNA-326 inhibitors led to a significant decrease in hepatic lipid accumulation, suggesting that miRNA-326 regulates hepatic lipid deposition. To explore the underlying mechanism, bioinformatic analysis was used to predict a direct interaction between miRNA-326 and FGF1 (Fig. 3C). A dual-luciferase reporter assay confirmed that co-transfection of miRNA-326 mimics with the wild-type FGF1 3’-UTR construct significantly reduced luciferase activity, whereas a mutant FGF1 construct showed no such change (Fig. 3D). Furthermore, transfection of miRNA-326 mimic resulted in significant downregulation of both FGF1 mRNA and protein expression (Fig. 3E, F), accompanied by a decrease in the levels of p-AMPK, while the total AMPK protein levels remained unchanged (Fig. 3E, F). In contrast, transfection with miRNA-326 inhibitors reversed these effects, restoring FGF1 expression and p-AMPK levels (Fig. 3E, F). These results suggest that miRNA-326 plays a crucial role in intrahepatic lipid accumulation by modulating the FGF1/AMPK signaling pathway.

### In vivo regulation of intrahepatic lipid accumulation by circZBTB46 and miRNA-326 via FGF1/AMPK

**Fig. 4:**
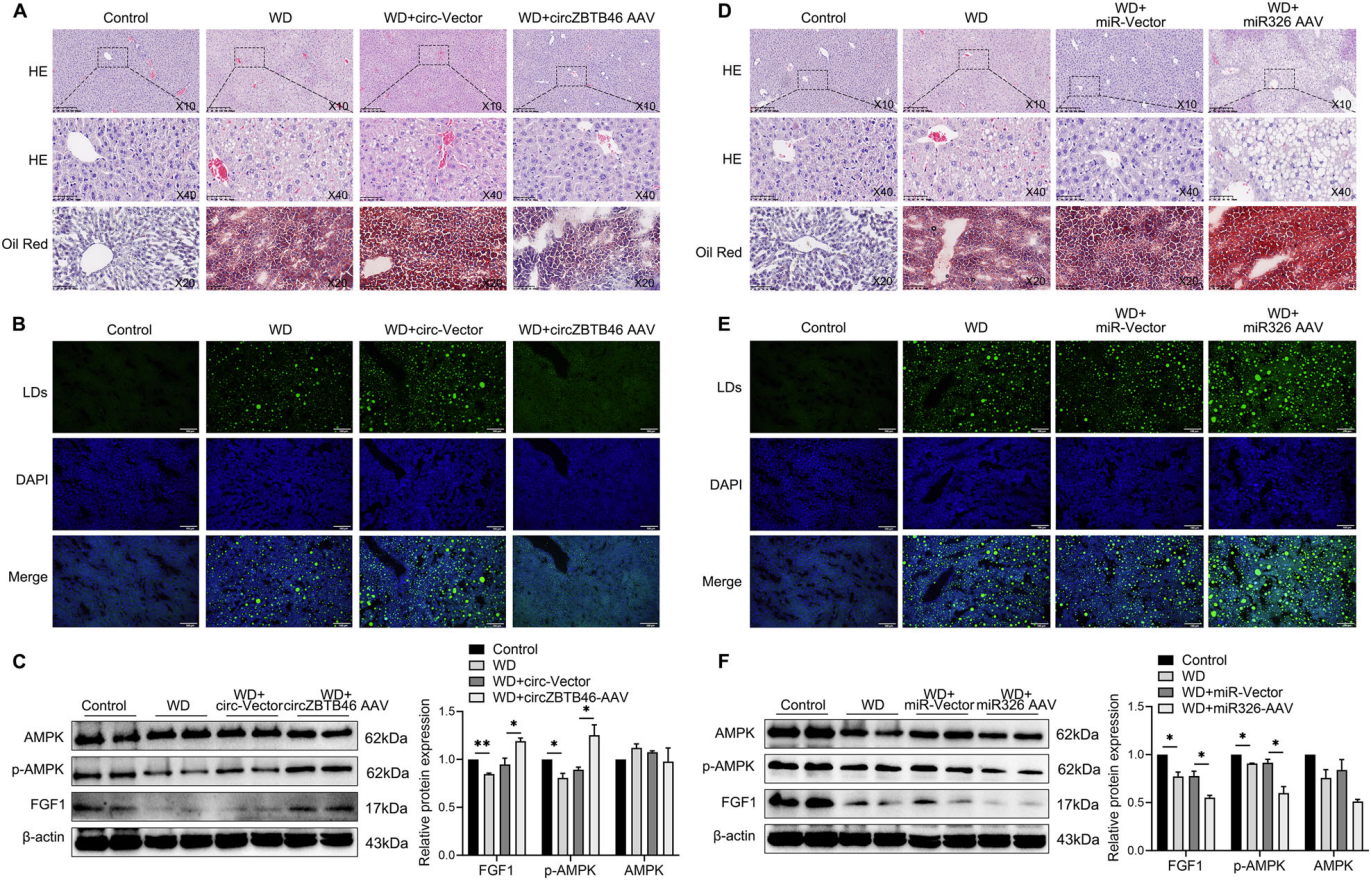
In vivo regulation of intrahepatic lipid accumulation by circZBTB46 and miRNA-326 via targeting FGF1/AMPK. **A** Representative images of H&E and Oil Red O staining of FFA-induced L02 cells transfected with circZBTB46 overexpression, interference, or control plasmids. Magnifications are H&E (10×, 40×) and Oil Red O (20×). **B** Representative images of BODIPY 493/503 staining in WD-fed mice treated with circZBTB46 AAV or control vector. Scale bars, 100 μm. **C** Western blot analysis and relative protein quantification assessed the expression of β-actin, FGF1, p-AMPK, and AMPK in liver tissues from WD-fed mice treated with circZBTB46 AAV or control vector. **D** Representative images of H&E (10×, 40×) and Oil Red O (20×) staining in WD-fed mice treated with miRNA-326 AAV or control vectors. **E** Representative images of BODIPY 493/503 staining in WD-fed mice treated with miRNA-326 AAV or control vectors. Scale bars, 100 μm. **F** Western blot analysis and relative protein quantification assessed the expression of β-actin, FGF1, p-AMPK, and AMPK in liver tissues from WD-fed mice treated with miRNA-326 AAV or control vector. Relative protein intensities were quantified using ImageJ software. Data represent mean ± S.D. Statistical significance was determined using a two-tailed unpaired Student’s t-test. *P < 0.05, **P < 0.01 compared to control.

To further investigate the in vivo roles of circZBTB46 and miRNA-326 in regulating hepatic lipid deposition via the FGF1/AMPK pathway, we administered intravenous injections of circZBTB46 AAV, miRNA-326 AAV, and their respective control AAV to mice. Administration of circZBTB46 AAV resulted in a significant decrease in intrahepatic lipid deposition, as shown by HE and Oil Red O staining (Fig. 4A). Furthermore, BODIPY 493/503 staining revealed a reduction in number and size of lipid droplets were observed through BODIPY 493/503 staining (Fig. 4B). These changes were accompanied by an increase in intrahepatic levels of FGF1 and p-AMPK (Fig. 4C). In contrast, intravenous administration of miRNA-326 AAV led to an increase in intrahepatic lipid deposition, as shown by HE and Oil Red O staining (Fig. 4D), and increased lipid droplet number, as observed by BODIPY 493/503 staining (Fig. 4E). Furthermore, miRNA-326 AAV administration resulted in reduced levels of FGF1 and p-AMPK in liver tissues (Fig. 4F). These results suggest that circZBTB46 and miRNA-326 exert opposing effects on intrahepatic lipid deposition by modulating the FGF1/AMPK signaling axis, thereby playing critical roles in the pathogenesis of MASLD.

### CircZBTB46 regulates FGF1/AMPK signaling and intrahepatic lipid deposition by competing with miRNA-326

**Fig. 5:**
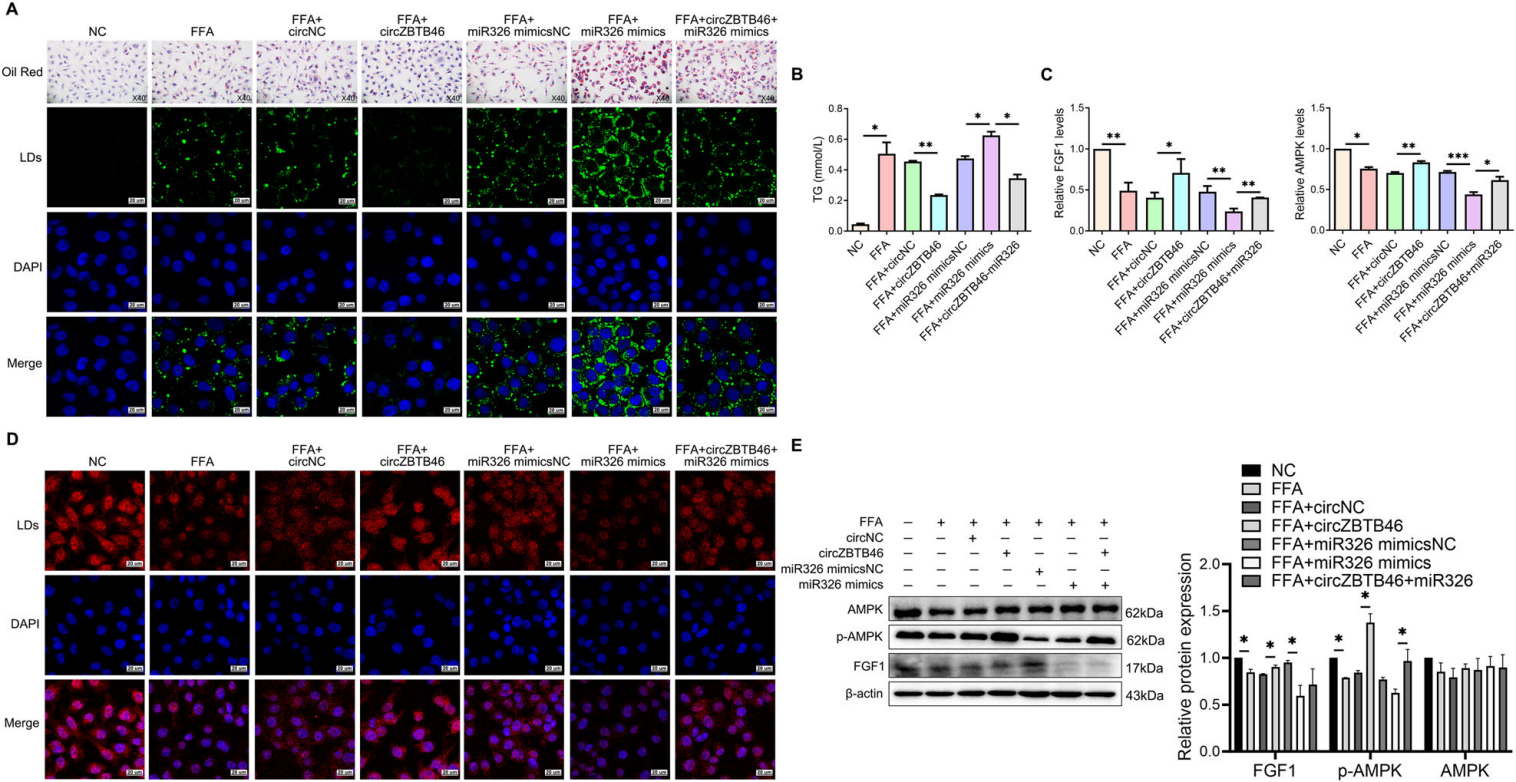
CircZBTB46 regulated FGF1/AMPK signaling via targeting miRNA-326 in vitro. **A** Representative images of Oil Red O (40×) and BODIPY 493/503 (scale bars = 20 μm) staining in FFA-induced L02 cells transfected or co-transfected with circZBTB46 overexpression plasmids, miRNA-326 mimics, and their matched controls. Lipid droplets (LDs) were stained with BODIPY 493/503 (green), and nuclei were stained with DAPI (blue). **B** Measurements of total triglyceride (TG) were conducted in FFA-induced L02 cells transfected or co-transfected with circZBTB46 overexpression plasmids, miRNA-326 mimics, and their matched controls. **C** qRT–PCR analysis assessed FGF1 and AMPK mRNA expression in FFA-induced L02 cells transfected or co-transfected with circZBTB46 overexpression plasmids, miRNA-326 mimics, and their matched controls. **D** Representative immunofluorescence images of FGF1 in FFA-induced L02 cells transfected or co-transfected with circZBTB46 overexpression plasmids, miRNA-326 mimics, and their matched control. Scale bars, 20 μm. **E** Western blot analysis and relative protein quantification assessed β-actin, FGF1, p-AMPK, and AMPK protein expression in FFA-induced L02 cells transfected or co-transfected with circZBTB46 overexpression plasmids, miRNA-326 mimics, and their matched control. Relative protein intensities were quantified using ImageJ software. Data represent mean ± S.D. Statistical significance was determined using a two-tailed unpaired Student’s t-test. *P < 0.05, **P < 0.01, ***P < 0.001 compared to control.

To investigate the role of circZBTB46 as a miRNA-326 sponge in regulating intrahepatic lipid deposition through the FGF1/AMPK pathway, we performed in vitro co-transfection experiments using circZBTB46 overexpression plasmids and miRNA-326 mimics. The results revealed that circZBTB46 led to a reduction in intracellular lipid deposition, as evidenced by Oil Red O and BODIPY 493/503 staining (Fig. 5A), and a decrease in triglyceride (TG) levels (Fig. 5B). In contrast, miRNA-326 promoted lipid accumulation (Fig. 5A, B). Notably, co-transfection with circZBTB46 and miRNA326 partially reversed the lipid reduction induced by circZBTB46, suggesting that miRNA-326 antagonizes the regulatory effects of circZBTB46 on lipid metabolism (Fig. 5A, B). Furthermore, qRT-PCR analyses showed that FFA downregulated the expression of FGF1 and AMPK, and miRNA-326 further decreased their expression. Conversely, circZBTB46 overexpression significantly upregulated FGF1 and AMPK levels, but this effect was attenuated when co-transfected with miRNA-326, suggesting that miRNA-326 modulates the lipid-reducing effects of circZBTB46 by counteracting its positive regulation of FGF1 expression (Fig. 5C). This finding was further validated by immunofluorescence and western blotting, which showed that circZBTB46 positively regulated FGF1 expression, while miRNA-326 exerted a negative regulatory effect (Fig. 5D, E). These results suggest that circZBTB46 acts as a competitive ceRNA for miRNA-326, alleviating its inhibitory effect on FGF1 expression. This interaction leads to an increase in the expression of FGF1, thereby reducing intrahepatic lipid deposition.

**Fig. 6:**
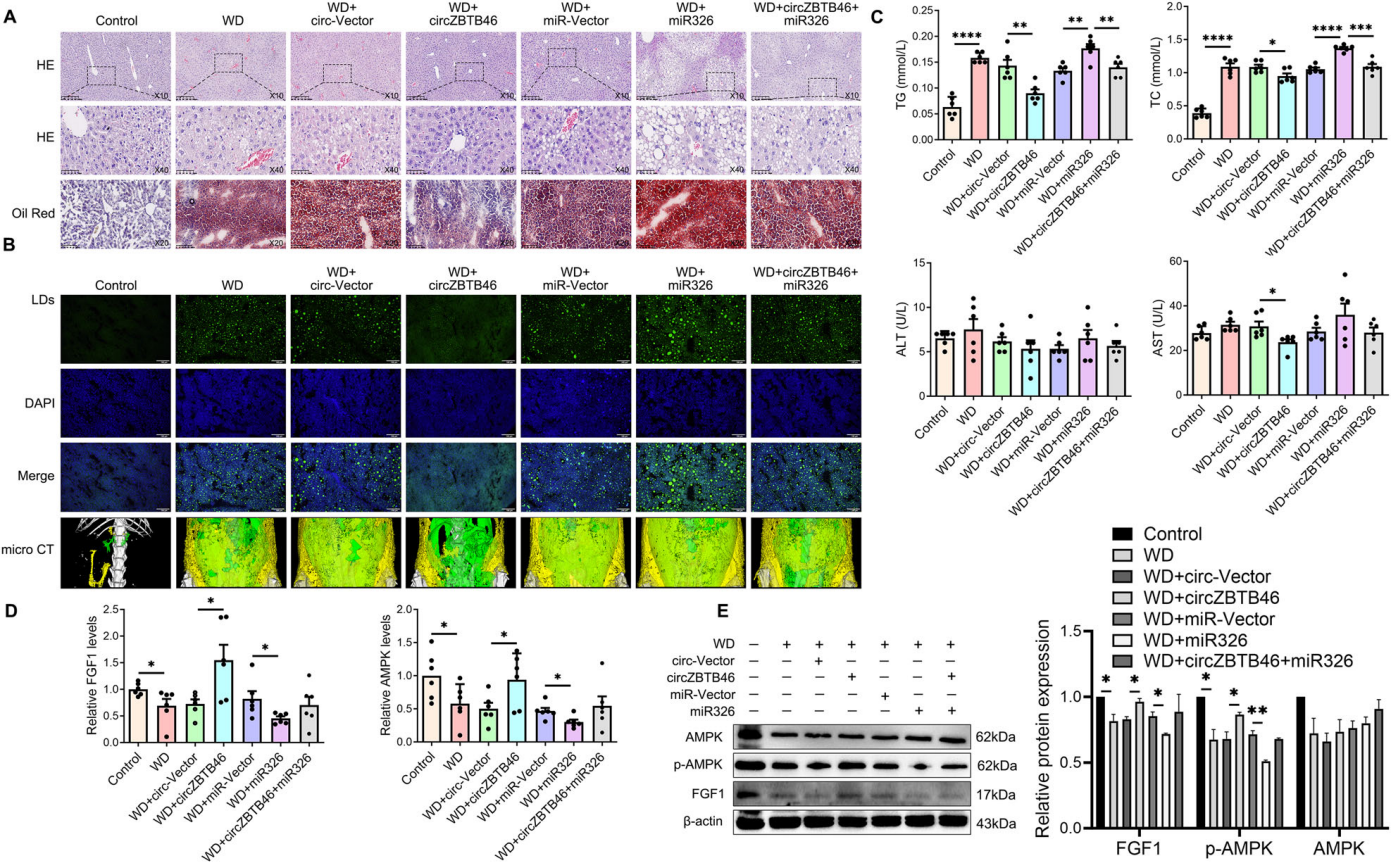
CircZBTB46 regulated FGF1/AMPK signaling via targeting miRNA-326 in vivo. **A** Representative images of H&E (10×, 40×) and Oil Red O (20×) staining in WD-fed mice treated with circZBTB46 AAV, miRNA-326 AAV, and their matched control vectors. **B** Representative images of BODIPY 493/503 (scale bars = 100 μm) staining and micro-CT imaging in WD-fed mice treated with circZBTB46 AAV, miRNA-326 AAV, and their matched control vectors. Lipid droplets (LDs) were stained with BODIPY 493/503 (green), and nuclei were stained with DAPI (blue). Micro-CT imaging was used to assess fat distribution, with green representing visceral fat and yellow indicating subcutaneous fat. **C** Measurements of triglycerides (TG), total cholesterol (TC), alanine aminotransferase (ALT), and aspartate aminotransferase (AST) levels were conducted in WD-fed mice treated with circZBTB46 AAV, miRNA-326 AAV, and their matched control vectors. **D** qRT–PCR analysis assessed FGF1 and AMPK mRNA expression in WD-fed mice treated with circZBTB46 AAV, miRNA-326 AAV, and their matched control vectors. **E** Western blot analysis and relative protein quantification assessed β-actin, FGF1, p-AMPK, and AMPK protein expression in WD-fed mice treated with circZBTB46 AAV, miRNA-326 AAV, and their matched control vectors. Relative protein intensities were quantified using ImageJ software. Data represent mean ± S.D. Statistical significance was determined using a two-tailed unpaired Student’s t-test. *P < 0.05, **P < 0.01, ***P < 0.001, ****P < 0.0001 compared to control.

**Fig. 7:**
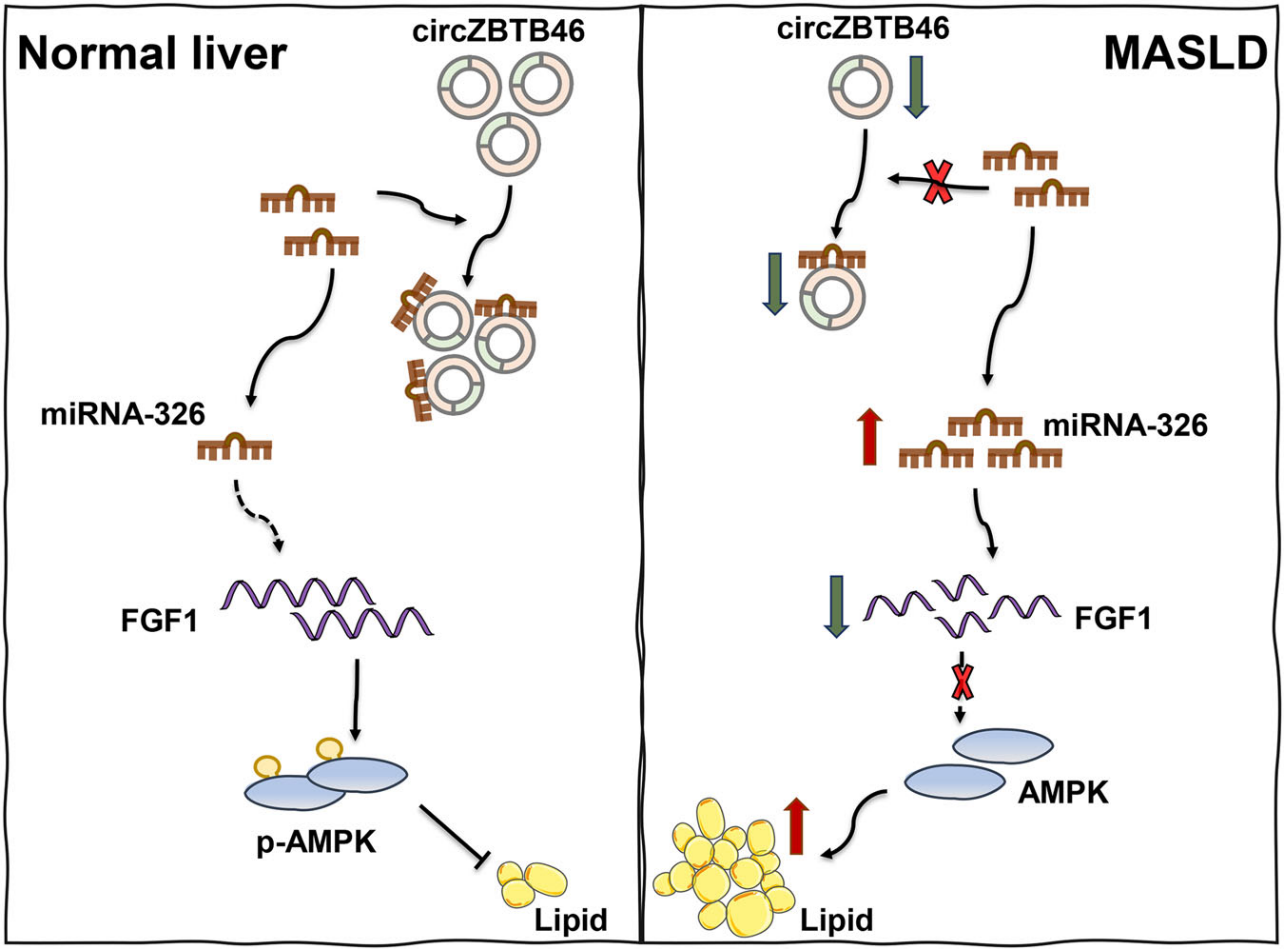
Schematic representation of the circZBTB46/miRNA-326/FGF1/AMPK signaling axis in MASLD pathogenesis. circZBTB46 functions as a competitive endogenous RNA (ceRNA) by sponging miRNA-326, preventing its suppression of FGF1 expression. This regulation sustains FGF1 levels, activating AMPK signaling and maintaining lipid homeostasis.

In the western diet-induced MASLD mouse model, we observed significant increases in intrahepatic lipid deposition, as evidenced by Oil Red O staining, HE staining, BODIPY 493/503 staining, and micro-CT imaging (Fig. 6A, B). Moreover, this diet resulted in increased hepatic levels of TG and total cholesterol (TC), and a decrease in the expression of FGF1 and AMPK (Fig. 6C–E). Furthermore, miRNA-326 AAV treatment exacerbated intrahepatic lipid accumulation and significantly suppressed the expression of FGF1 and p-AMPK (Fig. 6A–E). Conversely, circZBTB46 AAV treatment significantly decreased intrahepatic lipid deposition, lowered hepatic TG and TC levels, and increased the expression of intrahepatic FGF1 and p-AMPK (Fig. 6A–E). Notably, co-administration of circZBTB46 and miRNA-326 counteracted the beneficial effects of circZBTB46, resulting in a partial recovery of hepatic TG/TC levels and suppression of FGF1 and p-AMPK expression (Fig. 6A–E). These results suggest that circZBTB46 acts as a competitive sponge for miRNA-326, alleviating its post-transcriptional repression of FGF1, thereby activating the AMPK signaling pathway and improving intrahepatic lipid deposition. A schematic representation of this regulatory mechanism is shown in Fig. 7. This highlights the crucial role of the circZBTB46-miRNA-326-FGF1 signaling axis in the regulation of hepatic lipid metabolism.

## Discussion

The prevalence of metabolic dysfunction–associated steatotic liver disease (MASLD) is increasing, becoming a major global public health challenge. Due to its insidious onset and complex pathogenesis, early diagnosis and effective treatment of MASLD remain difficult, highlighting the need for innovative diagnostic approaches and therapeutic strategies. Circular RNAs (circRNAs) have emerged as promising biomarkers due to their widespread presence in bodily fluids and remarkable stability, offering new prospects for the early diagnosis and personalized treatment of MASLD [6]. This study is the first to systematically investigate the role of circZBTB46 in MASLD and clarify its molecular mechanism in regulating hepatic lipid metabolism through the miRNA-326/FGF1/AMPK signaling pathway. These findings enhance our understanding of circRNAs in metabolic diseases and provide potential molecular targets for early diagnosis and targeted treatment of MASLD.

Emerging evidence has established that circRNAs play crucial regulatory roles in MASLD by acting as competitive endogenous RNAs (ceRNAs) that sponge microRNAs (miRNAs) [11]. This study further revealed that circZBTB46 is downregulated in MASLD, and by binding to miRNA-326, it inhibits miRNA-326’s negative regulation of fibroblast growth factor 1 (FGF1). This interaction restores FGF1 expression and activates the AMPK signaling pathway, thereby alleviating lipid accumulation in MASLD. These findings provide novel mechanistic insights into the regulation of lipid metabolism in MASLD. miRNA-326 is involved in various cellular processes, such as cell proliferation, apoptosis, migration, invasion, chemoresistance in tumors, embryonic development, and immune regulation, highlighting its potential value in disease diagnosis and treatment [16, 17]. Yang et al. found that lncRNA Gm28382 promotes lipogenesis mediated by ChREBP through its interaction with miRNA-326-3p [18]. Furthermore, studies have shown that increased secretion of miRNA-326-3p from senescent adipose tissue exacerbates myocardial metabolic dysfunction in diabetic murine models [19]. In this study, the upregulation of miRNA-326 in MASLD was associated with increased hepatic lipid accumulation, further confirming the critical role of miRNA-326 in metabolic regulation. These findings suggest that further exploration of the functional mechanisms of miRNA-326 in MASLD could provide new insights into targeted therapeutic strategies for this disease.

FGF1 is a crucial metabolic hormone that regulates nutrient stress, maintains glycemic control, and improves insulin sensitivity. It plays a significant role in the treatment of metabolic diseases such as obesity and diabetes, and is increasingly recognized as a promising therapeutic target for diabetes management [20, 21]. The therapeutic potential of FGF1 in diabetes treatment also provides valuable insights for its potential clinical application in MASLD, especially given the significant progress made with FGF21—another member of the FGF1 family—in MASLD therapy and drug development [22]. In this study, we found that FGF1 expression was downregulated in MASLD. Functional experiments further revealed that miRNA-326 inhibits FGF1 expression, whereas circZBTB46 promotes its upregulation. When miRNA-326 and circZBTB46 were co-transfected, the positive regulatory effect of circZBTB46 on FGF1 expression was significantly suppressed and the reduction in lipid deposition mediated by circZBTB46 was reversed. This finding suggests that circZBTB46 modulates hepatic lipid accumulation in MASLD by competitively binding to miRNA-326, thereby alleviating miRNA-326-mediated suppression of FGF1. Interestingly, we observed a discrepancy between AMPK mRNA and protein levels. Although circZBTB46 and miRNA-326 affected AMPK mRNA levels, total AMPK protein levels remained stable, likely due to post-transcriptional regulatory mechanisms that maintain AMPK protein homeostasis. It is well established that AMPK activity is predominantly regulated by phosphorylation and dephosphorylation, rather than by changes in total protein expression. Consistent with this, our findings demonstrated that circZBTB46 and miRNA-326 significantly influenced AMPK phosphorylation, suggesting that they primarily regulate AMPK activity through modulation of its phosphorylation status. This finding is consistent with previous studies, such as those by Hu et al., who demonstrated that FGF1 improves lipid metabolism by promoting AMPK phosphorylation [23], and studies showing that a non-mitogenic variant of FGF1 can prevent and reverse liver steatosis and steatohepatitis by activating AMPK [15]. Our study revealed that circZBTB46 plays a crucial role in hepatic lipid metabolism by interacting with miRNA-326, thereby regulating FGF1 expression and AMPK phosphorylation. This regulatory axis holds significant therapeutic potential. Furthermore, recent studies by Li et al. developed a nanodrug system (GA-RM/GZ/PL) to specifically overexpress circRNA_0001805 in hepatocytes, effectively regulating lipid metabolism and inflammation to alleviate MASLD [24]. Compared to traditional viral vectors, nanoparticles and exosomes exhibit higher safety, ease of design, scalable production, efficient targeted delivery, superior biocompatibility, and stability. Therefore, exploring these innovative delivery systems to target circZBTB46 could significantly improve the efficacy and safety of circZBTB46-based therapies, providing new pathways for personalized treatment of MASLD.

This study has several limitations. First, we did not analyze the correlation between circZBTB46 expression and pathological changes in liver tissue. Although we observed a gradual decrease in its expression in MASLD and MASH patient samples, a more detailed histological examination is essential to clarify its cell-type-specific expression patterns and assess its association with inflammation or fibrosis progression. Second, the relatively small sample size of this study limited the comprehensive evaluation of circZBTB46 expression. Future research should include larger, multicenter, prospective cohorts encompassing patients with different etiologies (such as viral hepatitis, alcoholic liver disease, and autoimmune liver disease) and various disease stages. Furthermore, the use of patient-derived organoids or humanized PDX models could better recapitulate the pathophysiology of human MASLD and offer more accurate representations of the disease process. Multi-omics approaches, including single-cell sequencing and transcriptome-wide analyses, would be valuable for further dissection of the regulatory network of circZBTB46. Investigating circZBTB46 levels in easily accessible biological fluids, such as serum or urine, may offer valuable insights for non-invasive diagnosis. However, it is crucial to account for potential confounding factors, such as dietary habits, disease status, and medication use, which may influence circZBTB46 expression patterns. Finally, exploring the combination of circZBTB46 with other circRNAs or clinical parameters to build multimodal predictive models may enhance its clinical application value as a predictor of disease progression and prognosis.

## Conclusion

Our study revealed that circZBTB46 regulates hepatic lipid metabolism in MASLD by competitively binding to miRNA-326, thereby restoring FGF1 expression and activating the downstream AMPK signaling pathway. This study introduces a novel ceRNA-dependent mechanism by which circZBTB46 modulates MASLD progression, highlighting its dual role as a key driver of disease pathogenesis and a promising therapeutic target.

## Materials and methods

### MASLD patients and liver samples

Patients were diagnosed with MASLD according to the latest clinical guidelines, which require evidence of hepatic steatosis (confirmed by histology in our study) in conjunction with at least one cardiometabolic risk factor while excluding other known causes of liver diseases, such as viral hepatitis, autoimmune liver diseases, and excessive alcohol consumption [25]. Liver biopsies were performed on adult patients with MASLD (n = 3), MASH (n = 3), and healthy controls (n = 3) at West China Hospital of Sichuan University. Histological analyses were independently evaluated by two blinded pathologists. Specifically, patients with ≥5% hepatic steatosis without hepatocyte ballooning or other signs of hepatocyte injury were defined as having MASLD, while those with ≥5% hepatic steatosis accompanied by inflammation, hepatocyte ballooning, and other indicators of hepatocyte injury were classified as having MASH. This study protocol was approved by the Biomedical Ethics Committee of West China Hospital of Sichuan University, and written informed consent was obtained from all participants.

### Transcriptome sequencing

Transcriptome sequencing was performed by Novogene (Beijing, China). Total RNA was extracted from the patient’s liver tissues and assessed for quality. Following ribosomal RNA depletion and random fragmentation, strand-specific cDNA libraries were prepared using dUTP-based second-strand marking, adapter ligation, and size-selection. Libraries were quantified (Qubit), validated for insert size (Agilent 2100), and precisely quantified by qPCR before sequencing on an Illumina platform (PE 150 bp) using a sequencing-by-synthesis approach.

### Animals

Male C57BL/6 J mice, aged 6-8 weeks, were obtained from the Animal Experiment Center of Sichuan University. The mice were housed in a temperature-controlled environment with a 12/12-h light/dark cycle and allowed ad libitum access to food and water. After one week of acclimatization, the mice were assigned either a regular diet or a high-fat, high-sugar, and high-cholesterol Western diet (WD). The western diet, widely accepted for inducing steatosis in MASLD research [26], contained 0.15% cholesterol, 43% carbohydrates, 41% fat, and 17% protein, supplemented with high-sugar water, and was administered for 8 weeks (HFK BIOSCIENCE CO., LTD, China). Accordingly, the regular diet comprised 65.42% carbohydrates, 12.11% fat, and 22.47% protein, along with standard drinking water.

To achieve in vivo overexpression of circZBTB46 and miRNA-326, adeno-associated virus (AAVs) were used. Recombinant AAV vectors carrying circZBTB46 (HBAAV2/9-TBG-circZBTB46, 1.4 × 10^12^ vg/mL) and miRNA-326 (HBAAV2/9-TBG-miR326, 1.5 × 10^12^ vg/mL) were manufactured by Hanbio Co, Ltd (Shanghai, China). In the fourth week, AAVs and their corresponding empty vectors (1 × 10^12^ vg/mL) were intravenously injected. The mice were randomly divided into seven groups, with six mice per group: normal control (NC), WD, and five experimental groups receiving WD along with either circ-Vector, circZBTB46 AAV, miRNA-Vector, miRNA-326 AAV, or a combination of circZBTB46 and miRNA-326 AAVs. Four weeks after AAV delivery, overexpression of circZBTB46 and miRNA-326 was verified using immunofluorescence microscopy. Micro-CT was performed to measure the visceral and subcutaneous fat contents. After an 8-h fasting period, the mice were euthanized, and liver tissues and serum samples were collected for further analysis. All animal experiments were conducted in accordance with the guidelines of the Animal Care and Use Committee of Sichuan University and the National Research Council’s Guide for the Care and Use of Laboratory Animals.

### Cell culture and hepatic steatosis model induction

The normal human hepatocyte cell line L02, obtained from the Chinese Academy of Sciences (Shanghai, China), was cultured in RPMI-1640 medium (Gibco, USA) supplemented with 10% fetal bovine serum (FBS) (Gibco, USA), 100 U/mL penicillin, and 100 μg/mL streptomycin in a humidified incubator at 37 °C with 5% CO_2_. To establish a cellular model of MASLD, L02 cells were incubated for 24 h in complete RPMI-1640 containing 0.5 mM free fatty acids (FFA), a mixture of oleic acid and palmitic acid in a 2:1 volume ratio (Sigma-Aldrich, USA), as previously described [27].

### Plasmid construction and cell transfection

Plasmids targeting the junction region of circZBTB46 were constructed for knockdown and overexpression experiments. CircZBTB46 overexpression and shRNA plasmids, as well as miRNA-326 mimics and inhibitors, were obtained from GenePharma (Shanghai, China). Transfection was performed using the Lipofectamine 3000 reagent (Invitrogen, USA) according to the manufacturer’s instructions. Briefly, cells were cultured in 6-well plates until they reached 70–80% confluence (2 × 10^5^ cells/well), and then a mixture of plasmid (2 μg) and Lipofectamine 3000 (7.5 μL) was added to the medium. After 48 h of transfection, the transfected cells were collected for further experiments. The plasmid sequences are provided below:

miRNA-326 mimics: CCUCUGGGCCCUUCCUCCAG

miRNA-326 mimics NC: UUCUCCGAA CGUGUCACGUTT

miRNA-326 inhibitor: CUGGAGGAAGGGCCCAGAGG

miRNA-326 inhibitor NC: CAGUACUUUUGUGUAGUACAA

### Hematoxylin and eosin (H&E) staining

H&E staining was performed according to standard protocols. Briefly, mouse liver tissues were fixed in 4% paraformaldehyde for 24 h, embedded in paraffin, and sectioned (4 μm thickness). The sections were incubated in hematoxylin solution at room temperature for 20 min, washed with tap water, and differentiated in a 1% acid-ethanol solution. The sections were then stained with eosin solution for 30 s. Images of the tissue samples were acquired using a light microscope (OLYMPUS, Japan).

### Oil red O staining and BODIPY staining

Oil Red O and BODIPY staining were used to visualize lipid deposition in the cells and tissues. To assess lipid content, cultured cells and animal liver tissues were stained with Oil Red O staining (Sigma-Aldrich USA) following the manufacturer’s instructions. Briefly, cell slides or frozen liver tissue sections were incubated with Oil Red O staining solution for 10 min in the dark. Hematoxylin was used as a nuclear counterstain. After washing the slides with 60% isopropanol and distilled water, images were captured using an optical microscope (OLYMPUS, Japan). For BODIPY staining, the cells were washed with PBS and stained with BODIPY 493/503 for 15 min in the dark at 37 °C. Cells were then washed twice with PBS, fixed with 4% paraformaldehyde, washed three times with PBS for 5 min each, and stained with DAPI. The cells were imaged within 24 h using a confocal microscope (OLYMPUS, Japan). Tissue sections from fresh-frozen liver samples were prepared at a thickness of 10 μm and mounted onto microscope slides before undergoing the same staining procedure described above.

### Serum aminotransferase and lipid levels measurement

Total cholesterol (TC) and triglyceride (TG) levels were measured using a commercial ELISA kit (Nanjing Jiancheng Bioengineering Institute, China) according to the manufacturer’s protocols. ALT and AST levels were measured using an automatic biochemical analyzer (Hitachi, Japan).

### RNA extraction and quantitative real-time polymerase chain reaction (qRT-PCR)

Liver tissues or cultured cells were lysed using TRIzol reagent (Invitrogen, USA) according to the manufacturer’s protocol for total RNA extraction. The RNA concentration was quantified using a Nanodrop spectrophotometer (Thermofisher, USA). One microgram of extracted RNA was reverse-transcribed into cDNA using a TAKARA kit (Kusatsu, Japan) to serve as the template for PCR amplification. Genomic DNA (gDNA) was also extracted for subsequent analysis. qRT-PCR was performed using SYBR Green SuperMix (Vazyme Biotech, China) and the LightCycler®96 System (Roche Diagnostics GmbH, Germany), according to the manufacturer’s instructions. β-actin and U6 were used as internal controls for circZBTB46/mRNA and miRNA, respectively. The relative gene expression levels were calculated using the 2^-ΔΔCt method. The primer sequences used in this study were as follows:

CircZBTB46 (human): Forward primer: CGCTCATGAGTAAGAACAGCC, Reverse primer: GCCTCTTCTACAGACTGGGA;

CircZBTB46 (mouse): Forward primer: TGTTCGAGTACCTGCCCAAA, Reverse primer: GAGCGTCTTAAAGTAGCGGC;

β-actin (human): Forward primer: CTCCATCCTGGCCTCGCTGT, Reverse primer: GCTGTCACCTTCACCGTTCC;

β-actin (mouse): Forward primer: CAACTGGGACGACATGGA, Reverse primer: CCATCACAATGCCTGTGG;

For miRNA-326: Forward primer: GCGCCTCTGGGCCCTT, Reverse primer: AGTGCAGGGTCCGAGGTA;

U6: Forward primer: CTCGCTTCGGCAGCACA, Reverse primer: AACGCTTCACGAATTTGCGT.

### Western blot analysis

The samples were lysed in RIPA buffer (CST, USA) supplemented with PMSF. The lysates were mixed with SDS loading buffer (Solarbio, Beijing, China) and boiled for 5 min. Proteins were separated by sodium dodecyl sulfate-polyacrylamide gel electrophoresis (SDS-PAGE) and transferred onto polyvinylidene fluoride membranes. After blocking with 5% milk for 1 h, the membranes were incubated with specific primary antibodies overnight at 4 °C. Horseradish peroxidase-conjugated secondary antibodies (1:5000-10,000; Abcam, USA) were then incubated at room temperature for 1 h. Membrane exposure and visualization were performed using a chemiluminescent HRP substrate (Millipore, USA), and the images were captured using Bio-Rad Image Lab (Bio-Rad, USA). The primary antibodies used were as follows: Anti-FGF1 (1:1000; 17400-1-AP, Proteintech, China); Anti-p-AMPK (1:1000; 50081, Cell Signaling, USA); Anti-AMPK (1:1000; 5831, Cell Signaling, USA); Anti-β-actin (1:5000; TA-09, ZSGB-BIO, China).

### Actinomycin D and RNase R treatment

L02 cells were planted into six-well plates. Up to 60% confluency after 24 h, cells were treated with 5 μg/ml Actinomycin D or DMSO and collected at indicated time points. Total RNA (2 μg) was incubated with 3 U/μg of RNase R (Epicentre, USA) for 15 min at 37 °C. After treatment with Actinomycin D or RNase R, the RNA expression levels of circZBTB46 and ZBTB46 were analyzed by qRT-PCR.

### Luciferase reporter assay

Bioinformatic analyses (TargetScan, StarBase) were performed to identify putative miRNA-326 response elements in both circZBTB46 and the 3’ untranslated region (3’UTR) of FGF1. The wild-type (WT) sequences encompassing these predicted binding sites were synthesized and cloned into the GP-miRGLO dual-luciferase reporter vector (GenePharma, Shanghai, China). Corresponding mutant (MUT) constructs with altered miRNA-326 target sites were also generated. HEK293T cells were co-transfected with either WT or MUT reporter plasmids, together with miRNA-326 or negative control mimics, using Lipofectamine 2000 (Thermo Fisher, Waltham, MA, USA). Forty-eight hours after transfection, Firefly and Renilla luciferase signals were measured using the Dual-Luciferase Reporter Assay System (Promega, Madison, WI, USA). Relative luciferase activity was calculated by normalizing Firefly readings to Renilla signals, the latter serving as an internal control. Each experimental condition was performed in triplicate to ensure reproducibility.

### RNA pull-down

To investigate the interaction between circZBTB46 and miRNA-326, we performed an RNA pull-down assay using biotinylated circZBTB46 probes. HEK293T cells were transfected with a plasmid overexpressing circZBTB46, and after 48 h, approximately 1.5 × 10^7^ cells were collected and lysed under conditions that preserved RNA integrity. Biotinylated circZBTB46 and corresponding control probes were synthesized by Genepharm (Shanghai, China) and incubated with streptavidin magnetic beads (Thermo, USA). The cell lysates were then mixed with the probe-bound magnetic beads and incubated overnight at 4°C. Following extensive washes to remove non-specifically bound molecules, the captured RNA was extracted and analyzed using qRT-PCR.

### Statistical analysis

All quantitative data are presented as the mean ± standard deviation (SD). Statistical analyses were performed using GraphPad Prism 7 software. Relative protein intensities were quantified using ImageJ software. Differences between the two experimental groups were analyzed using Student’s t-test, while comparisons among three or more groups were assessed by one-way ANOVA followed by Tukey’s post hoc test. Statistical significance is indicated as *P < 0.05, **P < 0.01, ***P < 0.001, and ****P < 0.0001.

## Supplementary information

Supplementary information

## Data Availability

All data information used and/or analyzed during the current study is available from the corresponding author upon reasonable request.
