# Supplementary material for: CircZBTB46 alleviates metabolic dysfunction–associated steatotic liver disease by targeting miRNA-326/FGF1 axis
Source: Cell Death Discov. 2026 Jan 9;12:17. doi: 10.1038/s41420-025-02833-x (PMC12789582; doi:10.1038/s41420-025-02833-x)
Supplement: Supplementary file 1 — Supplementary information [file 41420_2025_2833_MOESM1_ESM.pdf]

Figure S1

A Cell

β-actin

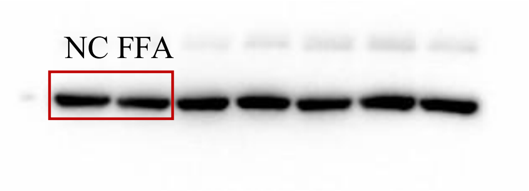

FGF1

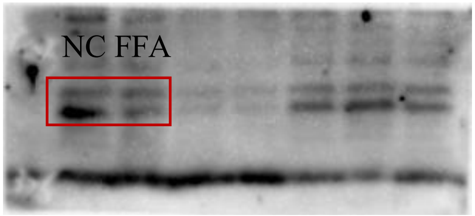

B Mice

β-actin

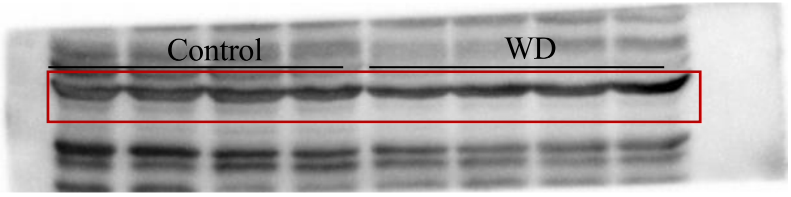

FGF1

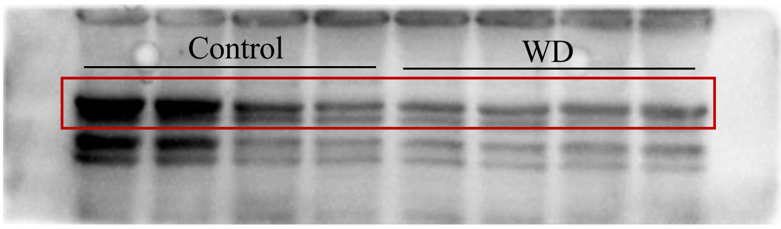

C Human

β-actin

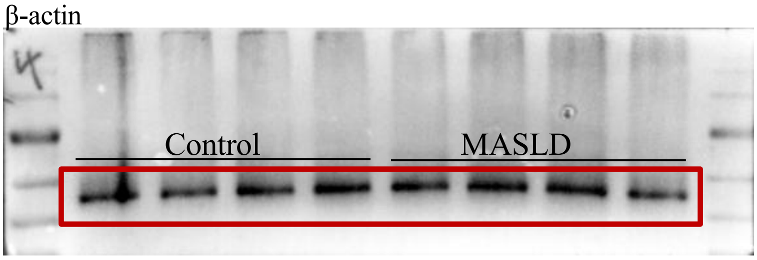

FGF1

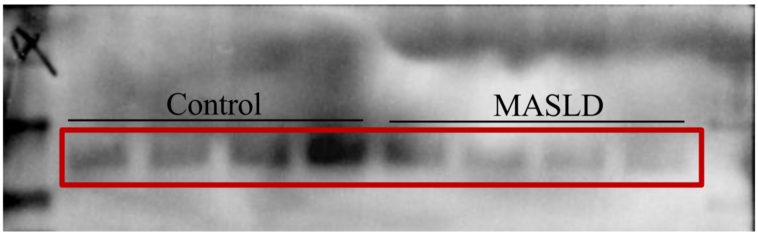

D

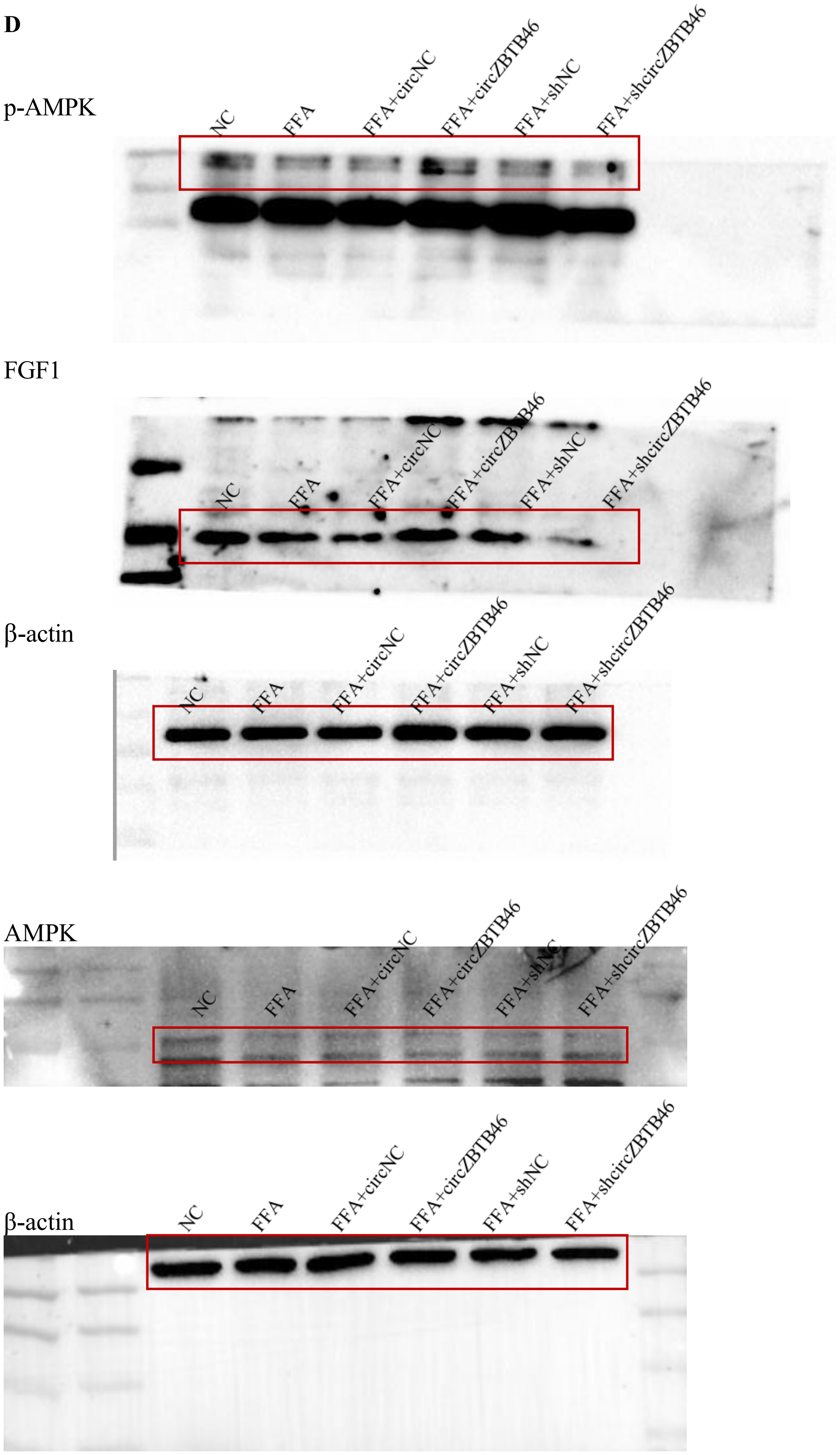

Figure S1 corresponds to the uncropped immunoblots shown in **Figure 2**. The membranes were cut with a scalpel to allow simultaneous detection of β-actin and FGF1 on the same membrane. Specifically, A corresponds to **Figure 2F**, showing the protein expression of β-actin and FGF1 in the FFA-induced MASLD cell model (Cell) and its control group. B corresponds to **Figure 2 F**, displaying the expression of β-actin and FGF1 in the WD diet-induced MASLD mouse model (Mice) and its control group. C corresponds to **Figure 2F**, depicting the protein levels of β-actin and FGF1 in MASLD patients (Human) and healthy controls. D corresponds to **Figure 2H**, illustrating the expression of β-actin, FGF 1, p-AMPK, and AMPK under different circZBTB46 levels. The bands used in **Figure 2** are marked with solid red boxes.

Figure S2

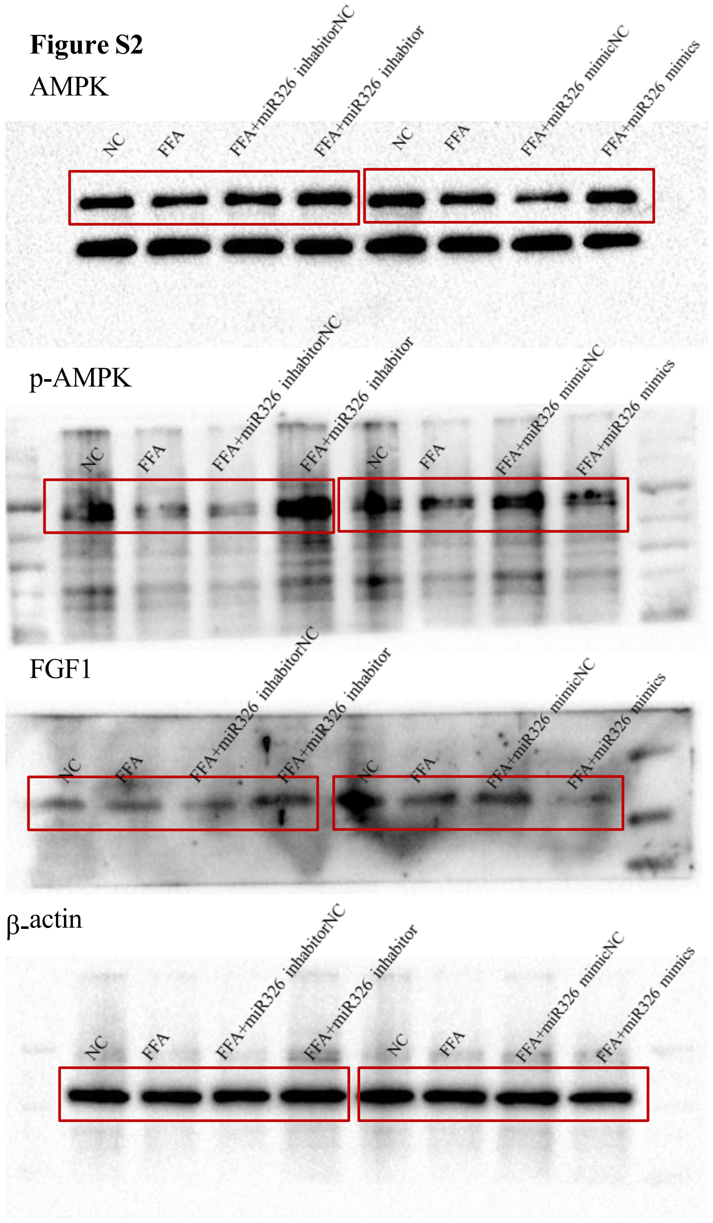

Figure S2 corresponds to the uncropped immunoblots in **Figure 3**, showing the protein expression levels of β-actin, FGF1, p-AMPK, and AMPK after in vitro transfection with miRNA-326 mimic and its control, as well as miRNA-326 inhibitor and its control. The membrane was cut with a scalpel to allow for simultaneous detection of β-actin, FGF1, p-AMPK, and AMPK on the same blot. The bands used in **Figure 3** are marked with solid red boxes.

Figure S3

A

β-actin

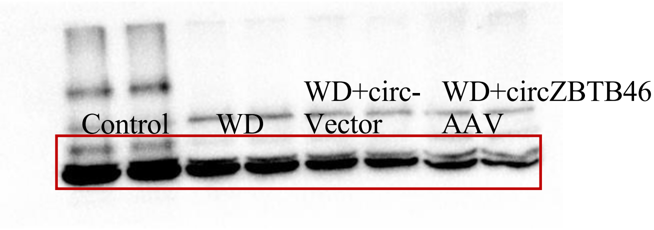

AMPK

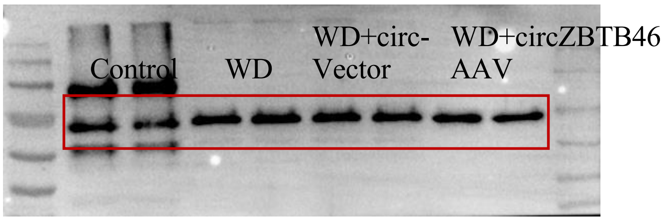

p-AMPK

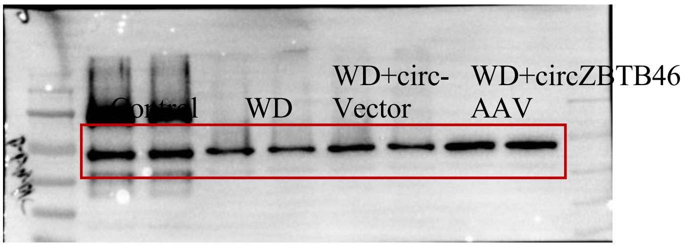

FGF1

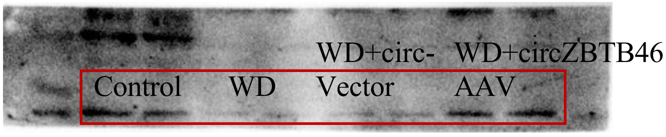

β-actin

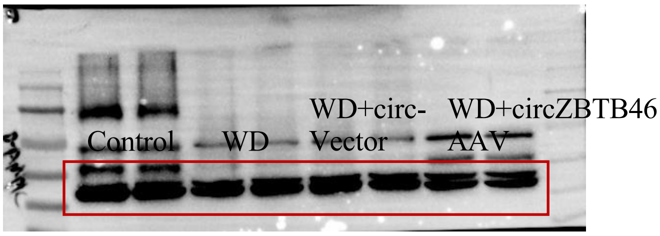

B

p-AMPK

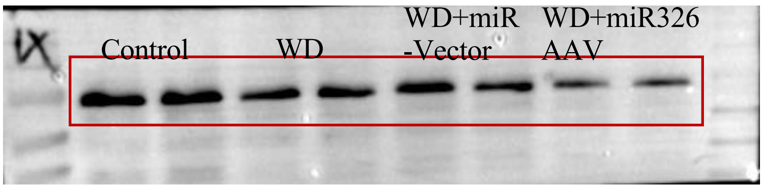

FGF1

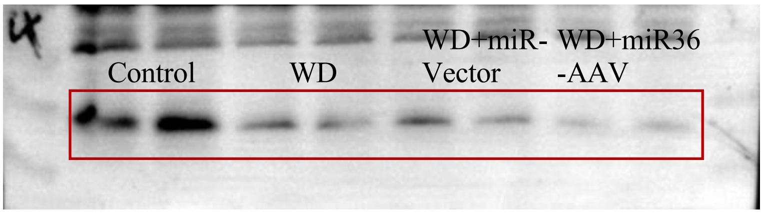

β-actin

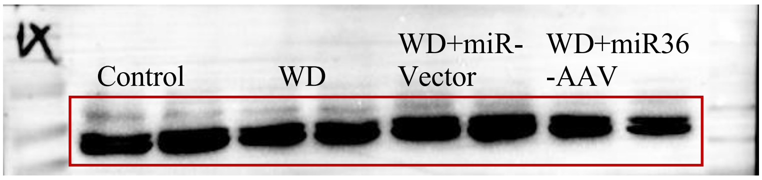

AMPK

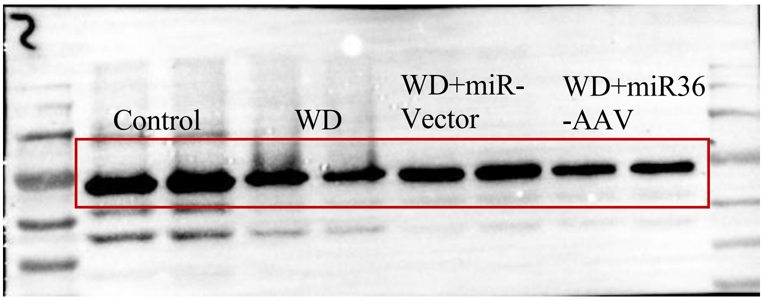

β-actin

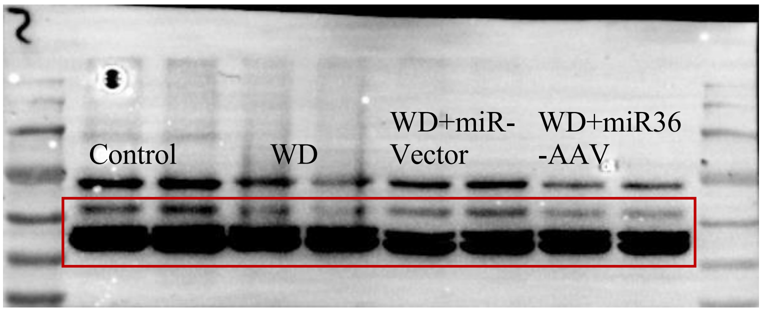

Figure S3 corresponds to the uncropped immunoblots shown in **Figure 4**. The membranes were cut with a scalpel to allow simultaneous detection of β-actin, FGF1, p-AMPK, and AMPK on the same membrane. A corresponds to **Figure 4C**, showing the changes in protein expression of β-actin, FGF 1, p-AMPK, and AMPK in WD diet-induced MASLD mouse models after intravenous injection of circZBTB46-overexpressing adeno-associated virus and its control. B corresponds to **Figure 4 F**, demonstrating the changes in protein expression of β-actin, FGF1, p-AMPK, and AMPK in WD diet-induced MASLD mouse models after intravenous injection of miRNA-326 adeno-associated virus and its control. The bands used in **Figure 4** are marked with solid red boxes.

Figure S4

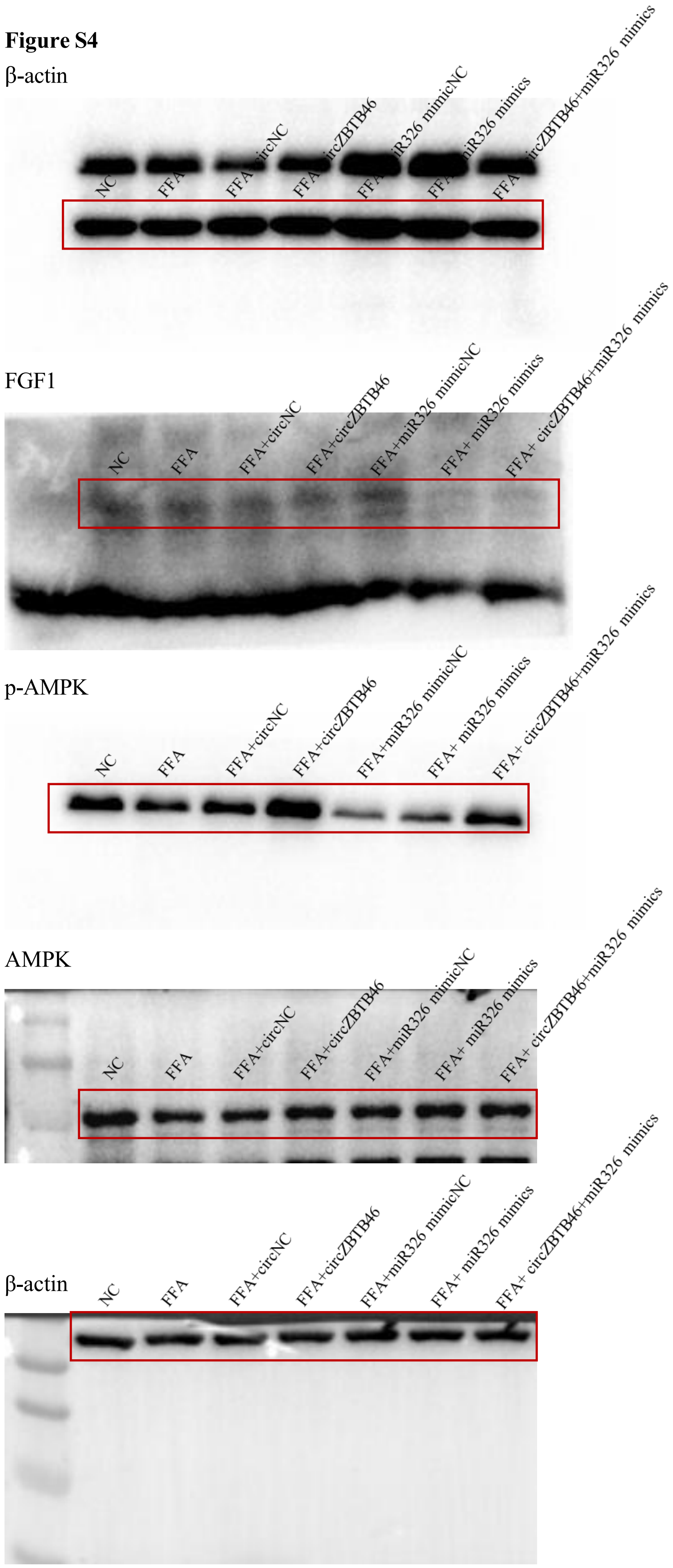

Figure S4 corresponds to the uncropped immunoblots in **Figure 5**, showing the protein expression levels of β-actin, FGF1, p-AMPK, and AMPK after transfection of circZBTB46 overexpression

plasmid and its control, miRNA-326 mimics and its control, as well as co-transfection of circZBTB46 overexpression plasmid and miRNA-326 mimics. The membranes were cut with a scalpel to allow for the simultaneous detection of β-actin, FGF1, p-AMPK, and AMPK on the same blot. The bands used in **Figure 5** are marked with solid red boxes.

Figure S5

AMPK

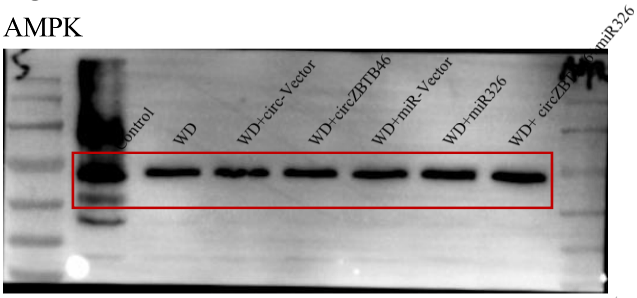

FGF1

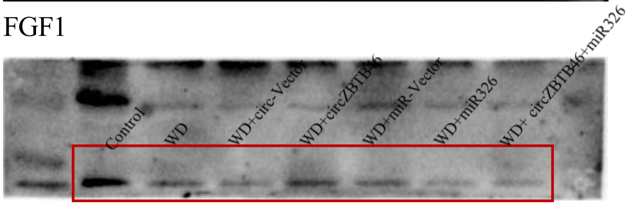

β-actin

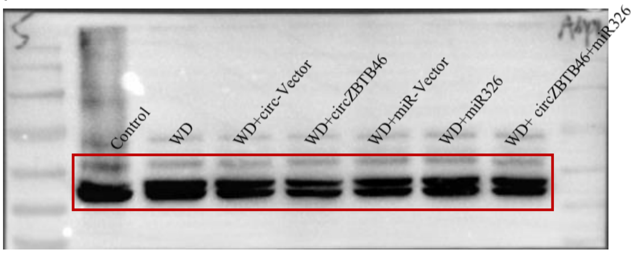

p-AMPK

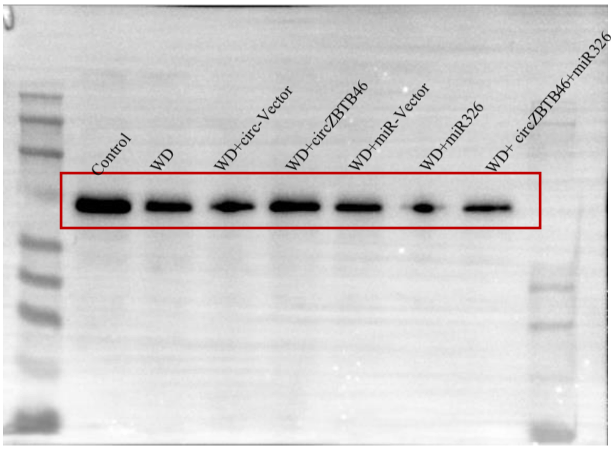

β-actin

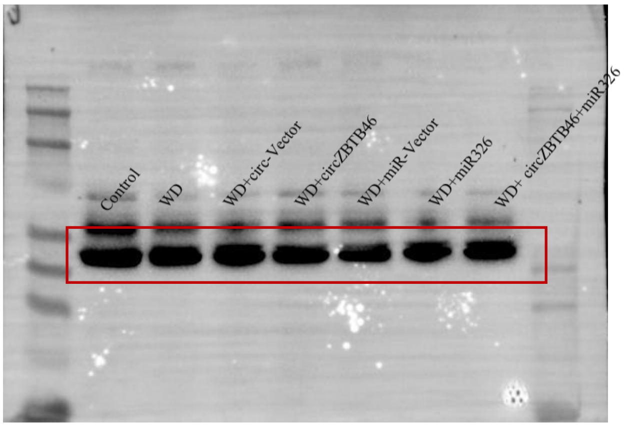

Figure S5 corresponds to the uncropped immunoblots in **Figure 6**, showing the protein expression levels of β-actin, FGF1, p-AMPK, and AMPK in liver tissues from mice with MASLD induced by the WD diet in vivo, following intravenous injection of adeno-associated viruses (AAVs) overexpressing circZBTB46 and their controls, miRNA-326 and their controls, as well as co-injection of AAVs overexpressing circZBTB46 and miRNA-326. The membranes were cut with a scalpel to allow for the simultaneous detection of β-actin, FGF1, p-AMPK, and AMPK on the same blot. The bands used in **Figure 6** are marked with solid red boxes.
